# 110. Improving Pneumococcal Vaccination in Patients Treated with Tumor Necrosis Factor-alpha Inhibitors: A Multidisciplinary Education-Based Quality Improvement Project

**DOI:** 10.1093/ofid/ofac492.188

**Published:** 2022-12-15

**Authors:** John M Curtin, Benjamin L Custer, Monique Norwood, Memar Ayalew, Varea H Costello, Dana M Blyth

**Affiliations:** Walter Reed National Military Medical Center, Rockville, Maryland; Walter Reed National Military Medical Center, Rockville, Maryland; Walter Reed National Military Medical Center, Rockville, Maryland; Walter Reed National Military Medical Center, Rockville, Maryland; Walter Reed National Military Medical Center, Rockville, Maryland; Walter Reed National Military Medical Center, Rockville, Maryland

## Abstract

**Background:**

In 2012, pneumococcal conjugate vaccine (PCV13) and pneumococcal polysaccharide vaccine (PPSV23) were recommended for immunocompromised adults ≥19 years, but pneumococcal vaccination (PV) in these patients (pts) remains suboptimal. With a new PV (PCV20) allowing for simplified PV, we study rates of PV in pts on infliximab (INX) 1 year post-initiation of a pt and provider education-based quality improvement project aiming to increase PV in pts on Tumor Necrosis Factor-alpha inhibitors (TNF-αI).

**Methods:**

Starting in 11/2020, pamphlets detailing PV indications were distributed to the Walter Reed National Military Medical Center infusion center and pharmacies to be given to pts prescribed/administered TNF-αI. Provider educational materials and pt pamphlets were given to services prescribing TNF-αI. Provider education was also offered to these services. Up to date (UTD) on PV was defined as receipt of PCV13 and PPSV23 (including additional PPSV23 dose ≥5 years post-initial PPSV23 if applicable). Ongoing INX (o-INX) was defined as INX initiation before 10/1/2020. Newly initiated INX (new-INX) was 10/1/2020-10/1/2021. One-year post implementation, we reviewed the PCV13 and PPSV23 immunization records of pts ≥19 years of age on INX from 1/1/21-12/31/21. This project was approved by the IRB under a non-research determination.

**Results:**

111 pts prescribed INX between 1/1/21-12/31/21 met inclusion criteria (87 o-INX and 24 new-INX). Prior to the intervention, of 87 o-INX pts, 45 (52%), 45 (52%), and 18 (21%) pts had received PCV13, ≥1 PPSV23 dose, and were UTD on PV respectfully. Between 1/1/21-12/31/21, 14 o-INX pts had PV (1 PCV13 and PPSV23, 7 PCV13, and 6 PPSV23), with 7 newly UTD (10% of the 69 previously not UTD), totaling 25 (29%) o-INX pts UTD on PV (figure 1). Of 24 new-INX pts, only 12 (48%), 9 (36%), and 6 (24%) had had PCV13, PPSV23, and were UTD on PV as of 12/31/21. No services prescribing TNF-αI requested the offered PV educational talks.

**Figure:**
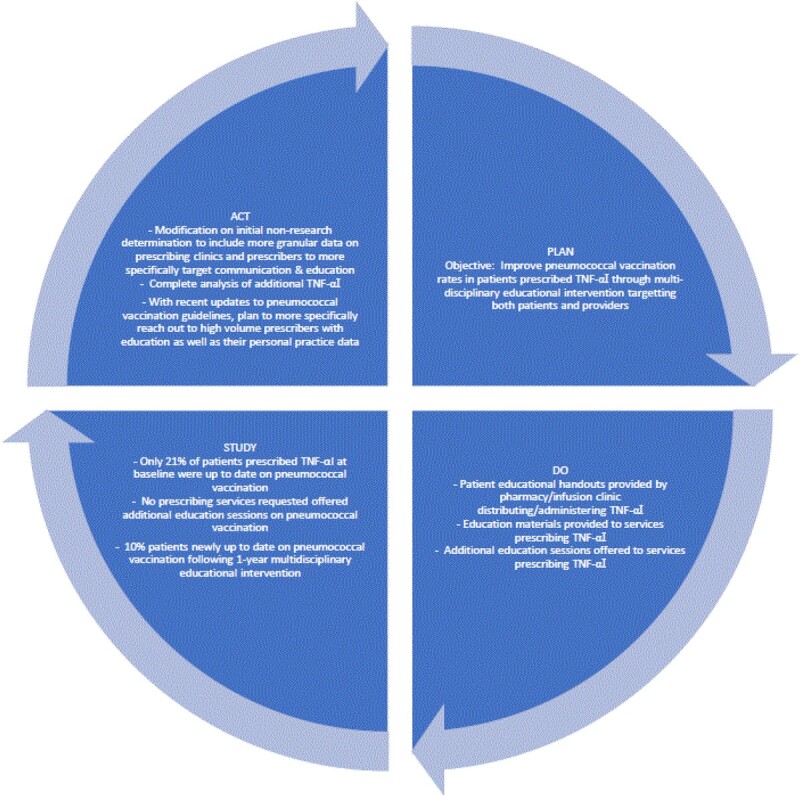

**Conclusion:**

Despite frequent healthcare in a system where vaccination has no out of pocket expense, guideline-concordant PV was low in this cohort. With new, simplified PV, next steps include updated pt and targeted provider education. However, with prior small gains using multidisciplinary education, additional efforts to remove PV barriers may be needed.

**Disclosures:**

**Benjamin L. Custer, MD**, Beam Therapeutics: Stocks/Bonds|CRISPR Therapeutics: Stocks/Bonds|glaxo-smith-kline: Stocks/Bonds|Hologic: Stocks/Bonds|Moderna: Stocks/Bonds|Pfizer: Stocks/Bonds|Regeneron: Stocks/Bonds|sanofi: Stocks/Bonds|Vertex Pharmaceuticals: Stocks/Bonds.

